# Pattern Transformation of Heat-Shrinkable Polymer by Three-Dimensional (3D) Printing Technique

**DOI:** 10.1038/srep08936

**Published:** 2015-03-11

**Authors:** Quan Zhang, Dong Yan, Kai Zhang, Gengkai Hu

**Affiliations:** 1 School of Aerospace Engineering, Beijing Institute of Technology, Beijing 100081, China

## Abstract

A significant challenge in conventional heat-shrinkable polymers is to produce controllable microstructures. Here we report that the polymer material fabricated by three-dimensional (3D) printing technique has a heat-shrinkable property, whose initial microstructure can undergo a spontaneous pattern transformation under heating. The underlying mechanism is revealed by evaluating internal strain of the printed polymer from its fabricating process. It is shown that a uniform internal strain is stored in the polymer during the printing process and can be released when heated above its glass transition temperature. Furthermore, the internal strain can be used to trigger the pattern transformation of the heat-shrinkable polymer in a controllable way. Our work provides insightful ideas to understand a novel mechanism on the heat-shrinkable effect of printed material, but also to present a simple approach to fabricate heat-shrinkable polymer with a controllable thermo-structural response.

Heat-shrinkable polymer has broad applications in various fields such as in intelligent medical devices^1^,^2^, packages for sensor and micro-systems^3^,^4^, and self-assembling systems^5^. In general, heat-shrinkable phenomenon of polymer relies on the release of internal stress/strain from two different fabricating steps: (i) processing step and (ii) programming step. Polymer, which is processed to obtain its original shape during processing step, will contract by annealing^6^. Afterward, the polymer can be deformed and fixed into a temporary shape under external stimuli, and then controllably cntracted into original shape upon heating, known as shape-memory phenomenon^3^. However, fabrication of heat-shrinkable polymer with controllable microstructures still remains a significant challenge. With development of three-dimensional (3D) printing technique, polymer with complex microstructure can be easily fabricated^7^,^8^,^9^,^10^,^11^,^12^,^13^,^14^,^15^,^16^,^17^,^18^ and the structure or microstructure of the printed sample can be achieved to deform under different stimuli by employing active printing materials such as shape memory polymer, called 4D printing^11^,^19^,^20^,^21^. However, internal stress/strain of the 4D-printed structures still need to be generated by applying external stimuli during programming step, not generated from processing step. It is reported that macro internal stress can be generated in polymer without the effect of phase transition during 3D printing process, and can result in structural shrinkage and distortion, or even fabrication failure^22^,^23^ at macroscale. No study has been reported on the exploration of the internal stress introduced from processing step to control the microstructural deformation of polymer under heating. In this work, we report the experimental observations of novel shrinkage and spontaneous pattern transformation of the polymer fabricated by 3D printing technique during heating, and then the underlying mechanism is revealed by evaluating the internal strain related to phase transition of the polymer. Finally, a simple approach is proposed to fabricate heat-shrinkable polymer which possesses controllable thermo-structural response and spontaneous pattern transformation under thermal stimuli.

## Results

### Experimental observation of novel shrinkage and pattern transformation of the printed polymer

**Figure 1:**
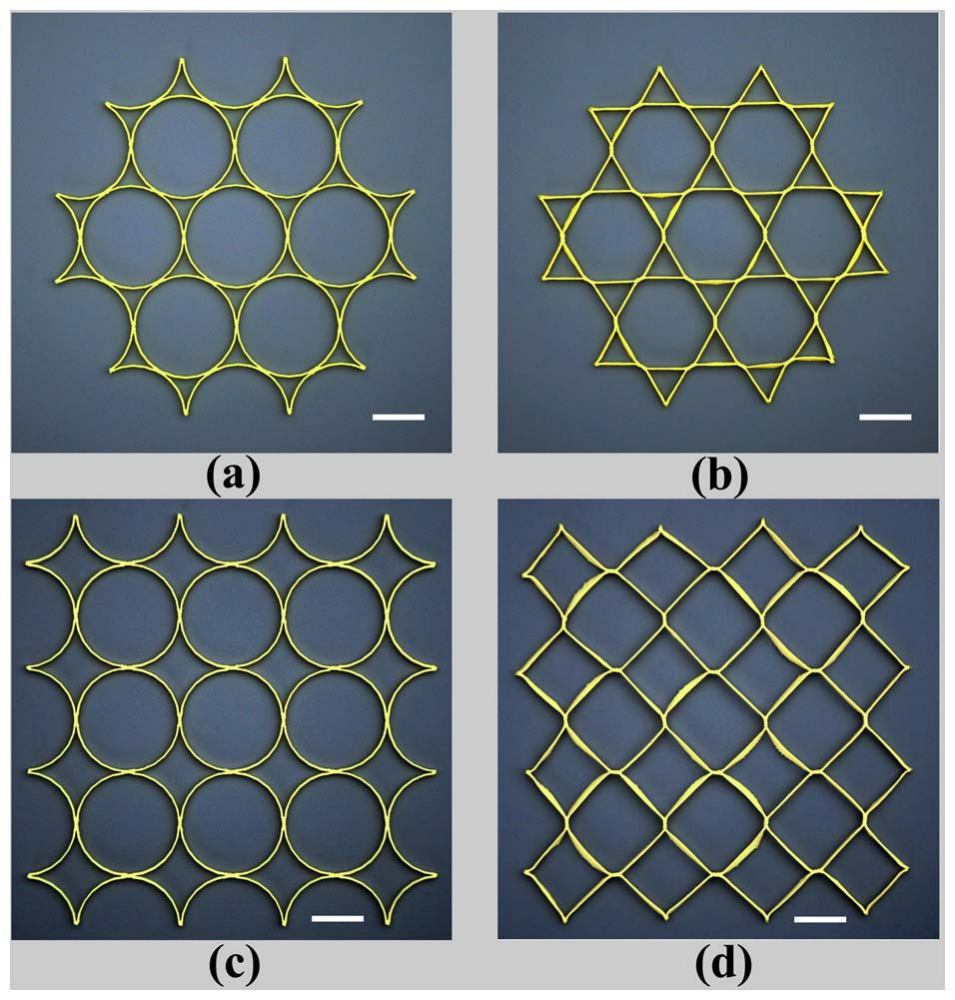
Pattern transformation of 2D lattice materials fabricated by 3D printing technique under heating. The 2D lattice materials, consisting of thin-walled PLA rings, are arranged initially in hexagonal pattern (a) or square pattern (c). The circular rings in the hexagonal or square lattices are transformed into hexagons (b) and quadrangles (d), respectively, when heated up to 90°C. Scale bar is 12 mm.

**Figure 2:**
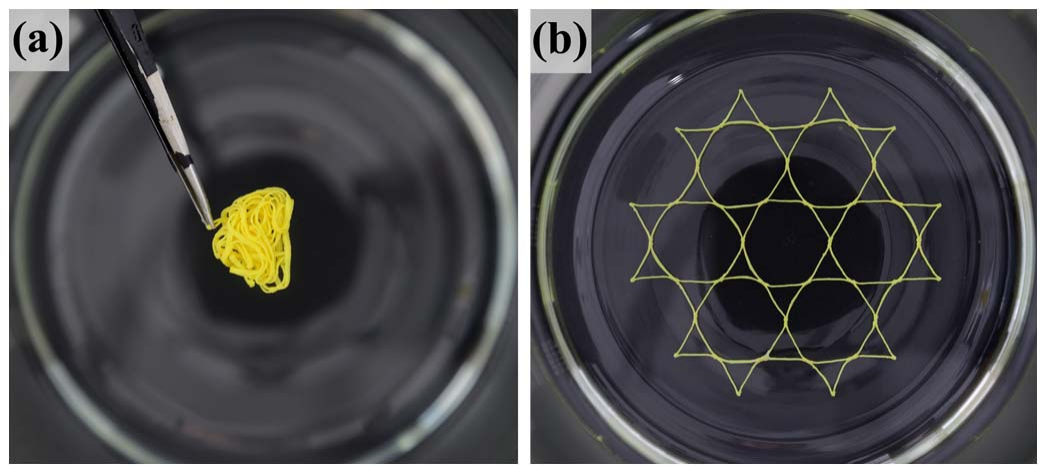
A depiction of the folding/unfolding process. (a) Temporary shape of the folded 2D lattice taken out from 70°C water; (b) Recovered original shape of the 2D lattice when plunged into 70°C water again, exhibiting a shape-memory effect.

Two dimensional (2D) lattice materials which were widely found in nature and engineering^10^,^13^,^24^,^25^,^26^,^27^, were studied here. Both the shape of single cell in lattice and the pattern arrangement were usually considered as the microstructure of the lattice materials. The designed 2D lattices consisted of thin-walled polylactic acid (PLA) rings with an outer diameter of 24 mm, thickness of 0.4 mm and height of 1.5 mm. The rings were arranged in hexagonal or square patterns and fabricated by 3D printing technique at a building speed of 90 mm s^−1^. Afterward, the printed lattice materials were heated on the surface of heating plate at 90°C. As shown in Fig. 1 (see Supplementary Movies 1 and 2), the entire lattices contracted at the beginning of heating, and then the circular rings in the hexagonal or square lattices were triggered into hexagons and quadrangles, respectively. It was also found that the lattice material arranged in the hexagonal pattern was transformed more rapidly to its final shape than that in the square pattern. When the lattice reached its final shape, it was immersed into a hot water of 70°C, and then folded roughly into a ball shape instantly after taken out of the hot water. As shown in Fig. 2 (see Supplementary Movie 3), the folded lattice was stable in room temperature, but can spread out immediately when plunged into the hot water again, exhibiting a typical characteristic of shape-memory effect. The shape-memory effect of PLA material has been reported in many previous literatures^19^,^28^,^29^,^30^. Therefore, the 3D-printed polymer, which presents a shape-memory effect, displays a heat-shrinkable characteristic and dramatical pattern transformation when heating.

### Underlying mechanism on thermal response of 3D-printed material

**Figure 3:**
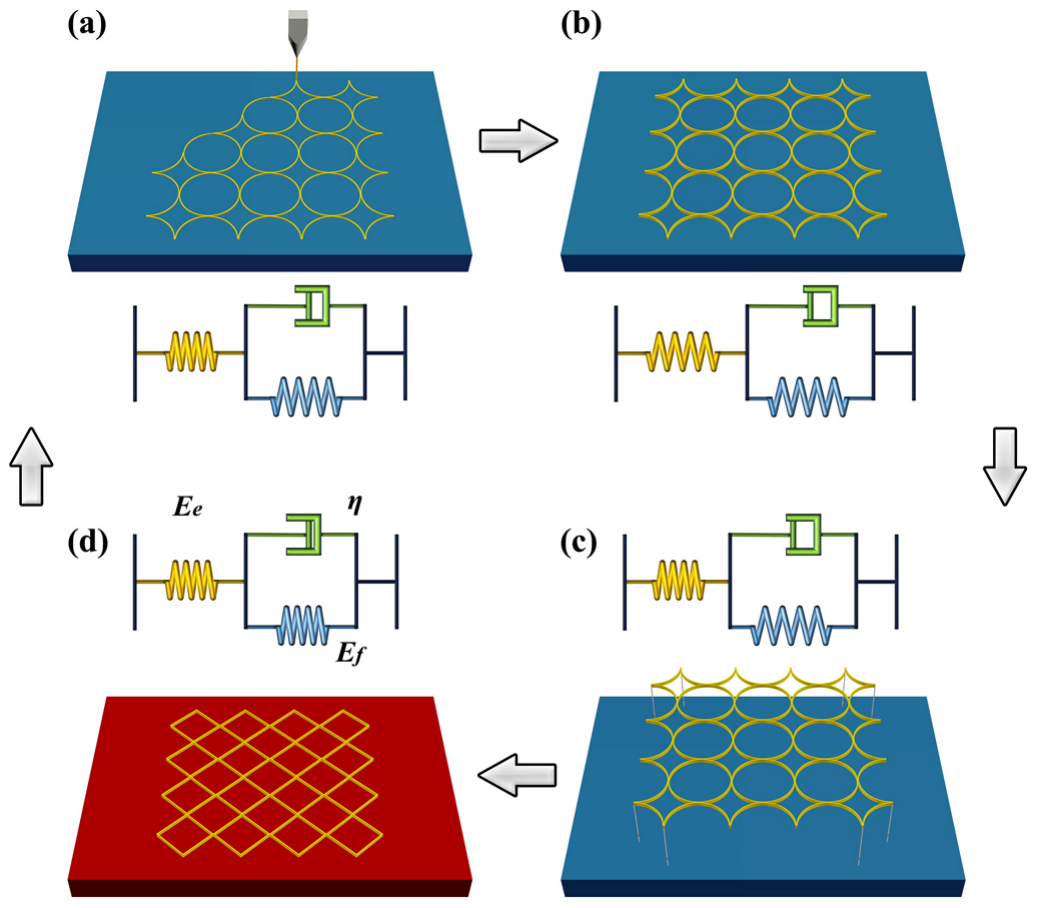
Schematic of fabrication process for 3D printing technique and the corresponding deformation of printed polymer described by a viscoelastic model. (a) The fused polymer is extruded from the nozzle and a constant strain is formed due to the moving of nozzle before it is bonded onto the platform of the 3D printer; (b) The printed polymer cools, solidifies, and bonds with platform or neighboring material and internal strain is generated during the process; (c) Removing the printed polymer from the platform leads to the recovery of elastic deformation, but an internal strain related to phase transition is stored in the printed polymer; (d) Internal strain stored in the polymer is released when reheated above its glass transition temperature, and can be explored to trigger pattern transformation of heat-shrinkable polymer.

The 3D printing technique used here is based on the fused deposition modeling (FDM), and its fabrication process is illustrated schematically in Fig. 3. The PLA material is firstly fused in the furnace of 3D printer, and then extruded from the nozzle. The printed material cools, solidifies, and bonds with platform or adjacent existing layers (Figs. 3(a) and 3(b)). During the fabricating process, the heating and rapid cooling cycles of the printed material will accumulate internal stress/strain due to the constraint of the platform or the existing layers. When removing the printed polymer from the platform (Fig. 3(c)), internal strain related to phase transition of the polymer can be stored for a long time, because the recovery of the polymer chains is prohibited below glass transition temperature (*T_g_*). By subsequent heating above *T_g_*, the stored strain can be released as the polymer chains are liberated, and can trigger shrinkage or pattern transformation of polymer (Fig. 3(d)).

**Figure 4:**
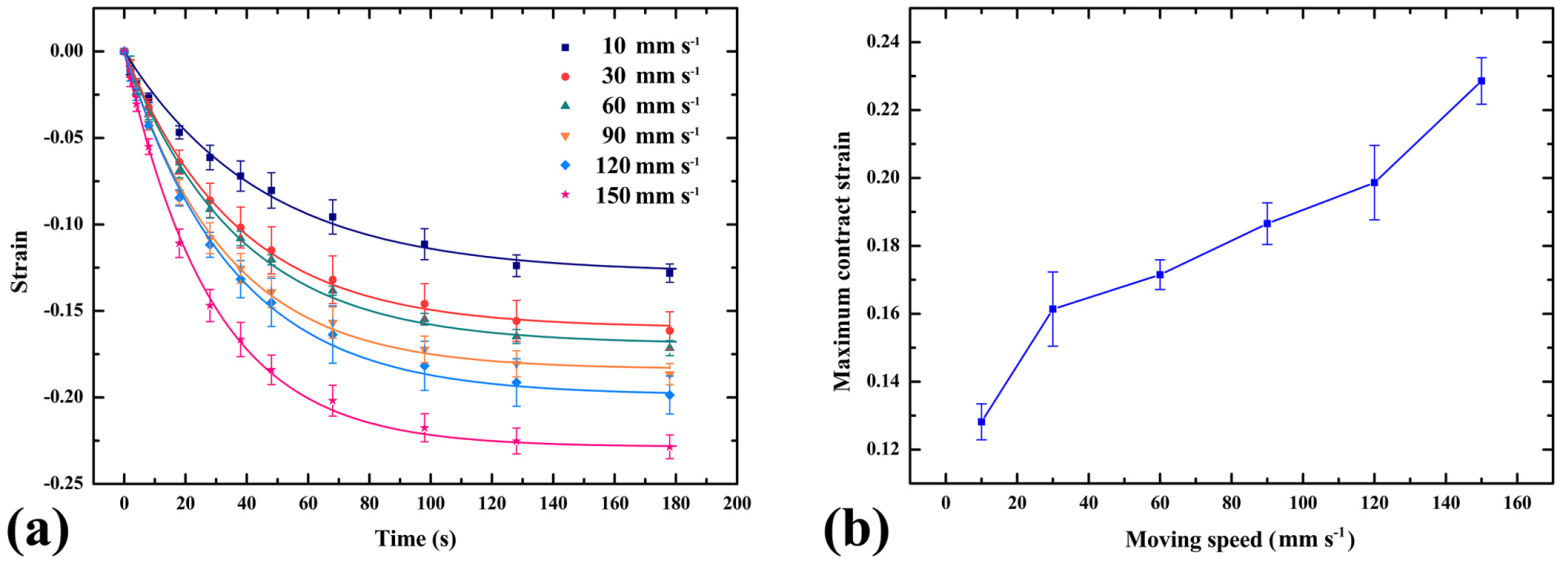
Experimental data and corresponding theoretical strain-time curves (a) and maximum contracted strains (b) for PLA strips at building speed varying from 10 to 150 mm s^−1^. All of the strips contract after heating and the maximum contract strain is almost linear to the building speed from 30 to 120 mm s^−1^.

In order to explain the observed thermo-mechanical property of the printed polymer, a simple structure of long strip is studied and a viscoelastic model is proposed, consisting of a classical Voigt model (a spring *f* (*E_f_*) and a dashpot (*η*) in parallel) and another spring *e* (*E_e_*) connected in series (Fig. 3). PLA strips are printed with the size of 20 × 1.6 × 0.6 mm (length × height × thickness) at building speeds of 10, 30, 60, 90, 120, 150 mm s^−1^, respectively. They were heated on the surface of a heating plate at 90°C. By calculating the ratio of the contraction to the initial length of the strips, we obtained the strains of the long strips at different time during the deformation process. More details on the experiment can be seen in method section and Supplementary Note. As shown in Fig. 4(a), all of the strips contracted after initial heating. The printed PLA material can shrink up to the maximum strain of 22.7% at the building speed of 150 mm s^−1^. We can also see that the maximum contracted strain is almost linear to the building speed *v* from 30 to 120 mm s^−1^, which is equal to 0.146 + 4.435 × 10^−4^*v* by linear fitting.

As the moving of nozzle at speed *v*, we assume that a short extruded PLA strip with length of Δ*x* is built with a constant strain *ε*_0_ before it is bonded onto the platform of the 3D printer (Fig. 3(a)). Subsequently, the material is cooled down from the nozzle temperature (*T_0_*) to *T_g_* (60°C for PLA material), and then to chamber temperature. The time for the first cooling process is 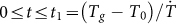, where the cooling rate (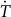) is assumed to be constant during the cooling process. The equilibrium equations are given as:
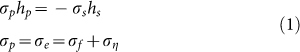
Where *h* is thickness and subscript *p* and *s* means the printed polymer and supporting platform, respectively. The constitutive relations in our model are given as:
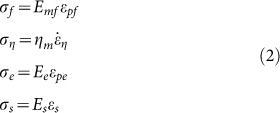
*E_mf_* and *η_m_* is the mean modulus and mean viscosity of spring *f*, respectively (see Supplementary Note). *E_e_* is the elastic moduli of spring *e* which can be assumed to be constant when the temperature is over its glass transition temperature. *E_s_* is the elastic modulus of the supporting platform and assumed to be constant during whole building process. The strain for the printed polymer is *ε_p_* = *ε_pe_* + *ε_pf_* = *ε_pe_* + *ε_η_*. So the consistent equation of the contact surfaces between platform and printed PLA material is given as:
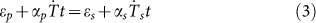
where *α_p_* and *α_s_* is the coefficient of thermal expansion of the printed polymer and supporting platform, respectively; 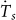 is the heating rate of platform. Then we can obtain the stress and strain of the printed material:
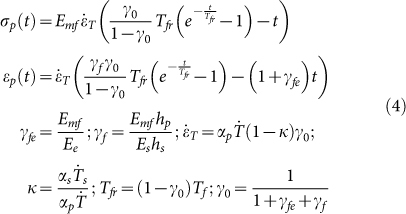
where the relaxation time *τ_f_* of PLA material is assumed to be constant over *T_g_*. For the second cooling process, the temperature of polymer is lower than the glass transition temperature, the material behaves as elastic material which is governed as linear elastic Hooke's equation. The strains during this process are given as:
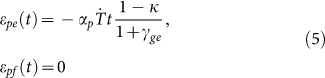
where *Y_ge_* = *E_ge_h_p_*/(*E_s_h_s_*) and *E_ge_* is the elastic modulus of the spring *e* below *T_g_*. After the material is cooled down to chamber temperature, the polymer chains are prohibited to deform and therefore the relaxation time at the glass state is very long. Removing the printed materials from the platform leads to the recovery of elastic deformation, that is, the strain kept in the spring *e* will recover completely while the strain in the spring *f* will be confined as shown in Fig. 3(c). Namely, an internal strain with the value of *ε_r_* retains in the spring *f* which is given as:
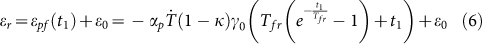
Therefore, the 3D-printed material can be considered to be a self-equilibrium state after being removed from the platform, where the spring *f* provides the recovery force while the dashpot provides the resistant force.

When the printed PLA strip is reheated with a heating rate 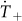, it will expand with a thermal strain 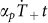 at beginning and then behave as a viscoelastic material once its temperature exceeds *T_g_*. At that state, the polymer chains are liberated and the strain related to phase transition is released. Since the spring *e* in the model has recovered completely, we simplify the system as a Voigt model, which consists of the spring *f* and the dashpot. The strain at temperature over *T_g_* can be given as:
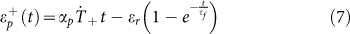
By neglecting the term 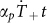, we use equation (7) to fit experimental results in Fig. 4(a) and obtain the relaxation time *τ_f_* and the internal strain *ε_r_*. We find that the average relaxation time is 35 ± 2 s and it varies little with the building speed, especially for range of 30 ~ 120 mm s^−1^. *ε_r_* shows a linear increase with the building speed, in accordance with experimental observation (Fig. 4(b)). Several researchers have reported the effect of 3D printing speed and temperature on material strength^22^,^23^,^31^. In our study, according to equation (6), the effect of the building speed on the contract strain is due to the variation on the constant strain *ε*_0_. The faster building speed can lead to a larger constant strain on the short PLA strip at the same time interval, when a short PLA strip Δ*x* is extruded from the nozzle, tensioned by the moving nozzle and completely bonded onto the platform of the 3D printer (Fig. 3(a)).

### Theoretical model of pattern transformation of printed cellular material

**Figure 5:**
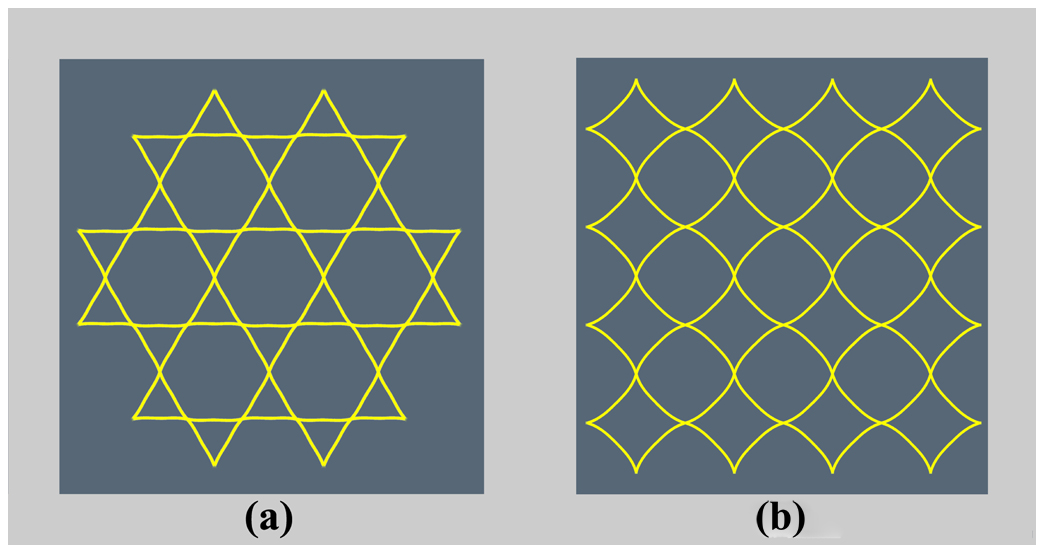
Calculated transformed pattern of the lattice materials arranged in hexagonal pattern (a) at 35 s or square pattern (b) at 70 s when heated to 90°C. The curved sides in the microstructures of 3D-printed polymer are transformed into straight lines, consistent with the experimental observations.

The pattern transformation of the lattice structures in Fig. 1 can also be predicted by our model. According to equation (7), a printed ring will contract when it is reheated initially over *T_g_*. The contraction of rings in the lattice can result in tension forces at the connected points between adjacent rings. However, the boundaries of the whole lattice structure are unconstrained. As a result, the assumed tension forces need to be released to zero quickly by structural motion or deformation. At the beginning of heating process, the polymer has a long relaxation time so that only spring *e* in our model can be stretched under the tension forces. The rings have large stiffness (modulus of spring *e*) to resist the deformation at that state, the circular rings therefore move toward the center of the structure until the tension forces vanish. The whole structure behaves as contraction without pattern transformation of circular microstructure. Later, polymer is gradually softened and moduli of spring *e* and *f* in the model drop dramatically. As the rings are easy to deform due to the reducing stiffness of the rings, the assumed tension forces induce the deformation of the circular rings. Afterward, viscosity of the material related to the dashpot is more significant than elasticity, which makes the deformed shape fixed instead of elastic recovery, and the novel patterns are finally formed. The deformed shapes of the circular rings varying with time can be described approximately as:
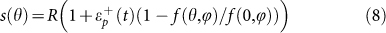
Where *f*(*θ*,*φ*) = (*φ* − *θ*)cos*θ − cosφsin(φ − θ)* is shape function, *θ* is polar coordinate of the circular ring in the lattice structure and *φ* describes the lattice structure of the material, that is, *φ* = *π*/6 and *π*/4 corresponds to hexagonal and square lattice structure, respectively; *R* is the radius of the rings in the lattice; 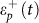 is given by equation (7). According to equation (8), the transformed shapes of the printed lattices are calculated and plotted in Fig. 5, and they are consistent with our experimental results (Figs. 1(b) and 1(d)). The triggering of pattern transformation is not dependent on the original printed microstructural shape and scale. For example, if the printed structure is arbitrary, not periodical, the pattern transformation still occurs. When the microstructure of the lattice materials is at micro-scale, the mechanism of pattern transformation is still at work. In addition, the effect of building speed on the final transformed shape can also be analyzed according to equations (7) and (8). For example, based on the theoretical analysis, we can find that, the shape can be transformed from circular rings into hexagons even at the building speed of 10 mm s^−1^, while it is difficult to transform circle rings in square lattice into quadrangles at low building speeds.

## Discussion

Conventional thermal-induced shape-memory polymer can also result in heat-shrinkage^3^. Compared with shape-memory polymer, internal strain stored in 3D-printed material is introduced from the processing step and caused by the constraint of platform, not by applying external forces on original shape during the programming step. The 3D printing technique generates a uniform distribution of internal strain in complex structures, which is hardly achieved by conventional manufacturing. As one of important advantages, the uniform internal strain in 3D-printed polymer enables the shape transformation of microstructure to be controlled accurately without the effect of non-uniform internal strain field. According to equation (6), the uniform internal strain can be further mediated by designing properties of platform, including CTE and modulus, temperate and heating rate, or applying external forces on the platform during the fabricating process. In addition, the proposed approach based on 3D printing technique can simplify the fabrication process of homogeneous shape-memory polymer, as well as achieve the fabrication of shape-memory polymer with tailored microstructures.

When printed polymer is subjected to thermal treatment, or applied in temperature-varied environment, thermal response of the printed polymer is of great importance for material or devices, especially for the precise microstructure of the material required in application. Furthermore, the demand on building speed of 3D printing technique necessarily causes increasing internal strain, which can't be eliminated by heating the platform or build chamber (equation (6)). The neglect of such inevitable heat-shrinkable phenomena caused by 3D-printing technique may lead to unexpected structural change. It is very necessary to develop the basic theoretical framework within which the effect of internal stress/strain from 3D-printing technique can be avoided. Besides the 3D printing technique based on the fused deposition modeling, there are many techniques under other mechanisms, such as droplet-based 3D printing and laser based printing technology^7^,^12^,^16^,^18^. The reported heat-shrinkable phenomenon and spontaneous pattern transformation of printed lattice materials are related to the uniform internal stress/strain, which is stored in printed shape memory polymer during the printing process and can be released under thermal stimuli. In essence, internal stress/strain can be generated by all of the 3D printing techniques. Thus, the reported phenomenon may be observed in other problems and corresponding theoretical models under different techniques need be studied further.

**Figure 6:**
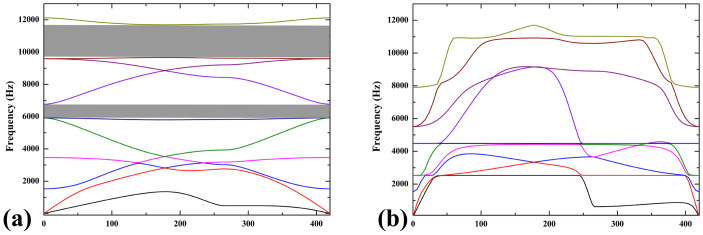
Frequency dispersion curves of the lattice materials with circular shape (a) and transformed hexagonal shape (b).

On the other hand, the beneficial internal stress/strain can be incorporated into design, which will innovate in the fabrication of the polymer material with microstructures. For example, as shown in Fig. 1, macro/micro-structural changing of printed polymer can also be achieved in a controllable way by monitoring the internal stress/strain induced in fabricating process. Meanwhile, it is reported that, the photonic and phononic properties and mechanical behaviors could be significantly altered due to pattern transformation of lattice material^24^,^32^,^33^,^34^,^35^. Our studies of the pattern transformations in lattices offer new insights into the manufacture and application of novel material fabricated by 3D printing technique. As shown in Fig. 6, the pattern transformations occurring under thermal stimuli strongly affect the band gaps of the material and wave directionality. For example, when the structure is transformed from the circular shape into hexagonal shape, the Bragg band gaps close. Our works can be exploited to the propagation of waves in some preferential directions or the considered frequency ranges^34^, leading to interesting applications for filtering, localizing and guiding acoustic waves.

## Conclusion

We investigate the thermal response of printed polymer and report a novel phenomenon for the 3D printing technique, namely, the printed polymer material has a heat-shrinkable property, whose initial microstructure can undergo a spontaneous pattern transformation under heating. The corresponding mechanism is due to the uniform internal stress stored in printed material, which is generated from 3D printing process and can be released under thermal stimuli. Our study offers new insights into the design, manufacture and application of the polymer with microstructures in engineering fields.

## Methods

### Material and experiments

The printed material in our work is MakerBot PLA Filament and supplied by MakerBot Company, whose glass transition temperature (*T_g_*) and melting temperature is about 60 ~ 65°C and 150 ~ 160°C, respectively. Mechanical properties of PLA including tensile modulus, tensile strength, and thermal analysis by using a differential scanning calorimetry (DSC) and dynamic mechanical analyses (DMA) can be found in other literatures^28^,^29^,^30^,^36^.

All the samples are printed by using a 3D polymer printer (MakerBot Replicator 2, MakerBot, Brooklyn, NY 11201 USA) with the highest resolution 0.1 mm per layer. The nozzle temperature is fixed to be 230°C and the building speed can be set from 10 to 150 mm s^−1^ for different samples.

As for the hexagonal and square lattice structures, the building speed is fixed to be 90 mm s^−1^ and the size of rings in lattices is designed to be an outer diameter of 24 mm, thickness of 0.4 mm and height of 1.5 mm.

Long PLA strips are printed with the size of 20 × 1.6 × 0.6 mm (length × height × thickness) and built at a sequence of building speeds of 10, 30, 60, 90, 120, 150 mm s^−1^ in order to explore the relationship between the strain and building speed. Afterwards, we put the printed samples on a heating plate with the temperature of 90°C and the ambient temperature is about 20°C. The deformation process under heating is recorded using a digital camera and by calculating the ratio of the contraction to the initial length of the strips from the video, we obtained the strains of the long strips at different time during the deformation process.

## Author Contributions

K.Z. proposed the key idea of this paper, conceived the experiments and developed the theoretical models. Q.Z. carried out the experiments and prepared most of the data. D.Y. assisted with the experiments. G.K.H. assisted with discussing the idea and results. All authors contributed to the writing of the paper.

## Supplementary Material

Supplementary Information Supplementary Note

Supplementary Information Supplementary Movie 1

Supplementary Information Supplementary Movie 2

Supplementary Information Supplementary Movie 3
