# Supplementary material for: Pattern Transformation of Heat-Shrinkable Polymer by Three-Dimensional (3D) Printing Technique
Source: Sci Rep. 2015 Mar 11;5:8936. doi: 10.1038/srep08936 (PMC4355736; doi:10.1038/srep08936)
Supplement: Supplementary Information — Supplementary Note [file srep08936-s1.pdf]

Supplementary Note

Pattern Transformation of Heat-Shrinkable Polymer by Three-Dimensional (3D) Printing Technique

Quan Zhang, Dong Yan, Kai Zhang & Gengkai Hu

School of Aerospace Engineering, Beijing Institute of Technology, Beijing 100081, China

Experimental contracted strains with different heating time for PLA strips

Long PLA strips are printed with the size of 20 × 1.6 × 0.6 mm (length × height × thickness). The long strips are built at a sequence of building speeds of 10, 30, 60, 90, 120, 150 mm s^{-1} in order to explore the relationship between the strain and building speed. Afterwards, we put the printed samples on a heating plate with the temperature of 90 ℃ and the ambient temperature is about 20 ℃. The deformation process under heating is recorded using a digital camera and by calculating the ratio of the contraction to the initial length of the strips from the video, we obtained the strains of the long strips at different time during the deformation process listed in Table 1.

Table1.Experimental contract strains with different heating time for PLA strips at building speed varying from 10 to 150 mm s^{-1}.

Time (s)	Strain					
	10mm/s	30mm/s	60mm/s	90mm/s	120mm/s	150mm/s
0	0	0	0	0	0	0
2	-0.011±0.002	-0.009±0.004	-0.007±0.005	-0.006±0.003	-0.013±0.004	-0.016±0.005
4	-0.019±0.003	-0.021±0.004	-0.021±0.004	-0.020±0.004	-0.025±0.003	-0.030±0.004
8	-0.027±0.003	-0.032±0.005	-0.037±0.003	-0.042±0.004	-0.043±0.002	-0.055±0.005
18	-0.047±0.004	-0.064±0.007	-0.069±0.004	-0.081±0.007	-0.085±0.005	-0.111±0.008

28	-0.061±0.007	-0.086±0.010	-0.091±0.005	-0.108±0.009	-0.112±0.007	-0.147±0.009
38	-0.072±0.009	-0.102±0.012	-0.108±0.004	-0.125±0.009	-0.132±0.011	-0.166±0.010
48	-0.080±0.010	-0.115±0.014	-0.120±0.003	-0.139±0.009	-0.145±0.014	-0.184±0.009
68	-0.096±0.010	-0.132±0.014	-0.138±0.003	-0.157±0.009	-0.164±0.017	-0.202±0.009
98	-0.111±0.009	-0.146±0.012	-0.155±0.003	-0.172±0.008	-0.182±0.014	-0.218±0.008
128	-0.124±0.006	-0.156±0.012	-0.165±0.004	-0.181±0.008	-0.191±0.014	-0.225±0.007
178	-0.128±0.005	-0.161±0.011	-0.171±0.004	-0.187±0.006	-0.199±0.011	-0.229±0.007

Theoretical model for thermal response of 3D printed material

By considering a simple structure of long strip, a viscoelastic model consisting of a classical Voigt model (a spring f (E_f) and a dashpot (η) in parallel), and another spring (E_e) connected in series, is proposed to explain the mechanism of thermal response of the 3D printed material (Fig. S1).

The PLA material is firstly fused in the furnace of 3D printer, and then extruded from the nozzle. As the moving of nozzle at speed v, we assume that a short extruded PLA strip with length of Δx is built with a constant strain ε_0 before it is bonded onto the platform of the 3D printer (Fig. S1(a)). As the printed material is semi-solid state, the corresponding strains of the spring e and f can be simplified to be $\varepsilon_{pe} = 0$ and $\varepsilon_{pf} = \varepsilon_0$.

Subsequently, the material is cooled from the nozzle temperature T_0 to the glass transition temperature T_g (60 ℃ for PLA material). The time for this cooling process is $0 \le t \le t_1 = (T_g - T_0)/\dot{T}$, where the cooling rate ($\dot{T}$) is assumed to be constant during the cooling process. The equilibrium equations are given as:

$$\begin{aligned} \sigma_p h_p &= -\sigma_s h_s \\ \sigma_p &= \sigma_e = \sigma_f + \sigma_\eta \end{aligned} \quad (1)$$

where h is thickness and subscript p and s means the printed polymer and supporting platform, respectively. The strain for the printed polymer is $\varepsilon_p = \varepsilon_{pe} + \varepsilon_{pf} = \varepsilon_{pe} + \varepsilon_{\eta}$. According to Ref. 1, the viscosity of polymer fits the WLF equation:

$$\eta(t) = \eta_g e^{\frac{-17.4(T-T_g)}{51.6+T-T_g}} \tag{2}$$

where η_g is the viscosity of polymer at glass transformation temperature. We define the mean viscosity and mean modulus of spring f:

$$\eta_m = \frac{1}{t}(\int_0^t \eta(t')dt') = \frac{\eta_g}{\Delta T}(\int_{T_0}^{T_0+\Delta T} e^{\frac{-17.4(T'-T_g)}{51.6+T'-T_g}} dT') \tag{3}$$

$$E_{mf} = \frac{\eta_m}{\tau_f} = \frac{\eta_g}{\tau_f \Delta T}(\int_{T_0}^{T_0+\Delta T} e^{\frac{-17.4(T'-T_g)}{51.6+T'-T_g}} dT') \tag{4}$$

E_e and τ_f can be assumed to be constant when the temperature is over its glass transition temperature. The constitutive relations in our model are given as:

$$\begin{aligned} \sigma_f &= E_{mf}\varepsilon_{pf} \\ \sigma_\eta &= \eta_m \dot{\varepsilon}_\eta \\ \sigma_e &= E_e \varepsilon_{pe} \\ \sigma_s &= E_s \varepsilon_s \end{aligned} \tag{5}$$

where E_s is the elastic modulus of the supporting platform and assumed to be constant.

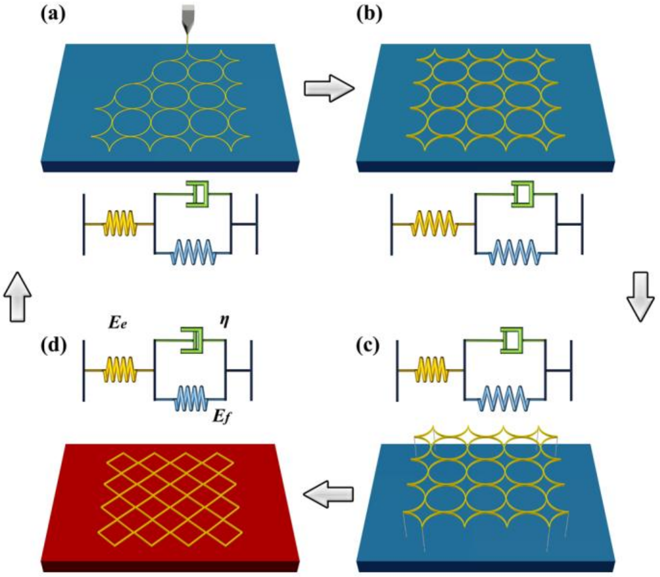

Figure S1 Schematic of fabrication process for 3D printing technique and the corresponding deformation of printed polymer described by a viscoelastic model. (a) The fused polymer is extruded from the nozzle and a constant strain is formed due to the moving of nozzle before it is bonded onto the platform of the 3D printer; (b) The printed polymer cools, solidifies, and bonds with platform or neighboring material and internal strain is generated during the process; (c) Removing the printed polymer from the platform leads to the recovery of elastic deformation, but an internal strain related to phase transition is stored in the printed polymer; (d) Internal strain stored in the polymer is released when reheated above its glass transition temperature, and can be explored to trigger pattern transformation of heat-shrinkable polymer.

During the cooling process from T_0 to temperature T_g, consistent equation of the contact surfaces between platform and printed PLA material is given as:

$$\varepsilon_p + \alpha_p \dot{T} t = \varepsilon_s + \alpha_s \dot{T}_s t \tag{6}$$

where α_p and α_s is the coefficient of thermal expansion of the printed polymer and supporting platform, respectively; $\dot{T}_s$ is the heating rate of platform. The initial condition is $\sigma_p(t)\big|_{t=0} = 0$, when $\gamma_f = \frac{E_{mf}}{E_s}\frac{h_p}{h_s} \neq 1$, we can obtain the stress and strain of the printed material:

$$\sigma_p(t) = E_{mf}\dot{\varepsilon}_T(\frac{\gamma_0}{1-\gamma_0}\tau_{fr}(e^{-\frac{t}{\tau_{fr}}} - 1) - t)$$

$$\varepsilon_p(t) = \dot{\varepsilon}_T(\frac{\gamma_f\gamma_0}{1-\gamma_0}\tau_{fr}(e^{-\frac{t}{\tau_{fr}}} - 1) - (1+\gamma_{fe})t) \tag{7}$$

$$\gamma_{fe} = \frac{E_{mf}}{E_e}; \gamma_f = \frac{E_{mf}h_p}{E_s h_s}; \dot{\varepsilon}_T = \alpha_p\dot{T}(1-\kappa)\gamma_0; \kappa = \frac{\alpha_s\dot{T}_s}{\alpha_p\dot{T}}; \tau_{fr} = (1-\gamma_0)\tau_f; \gamma_0 = \frac{1}{1+\gamma_{fe}+\gamma_f}$$

where the strains in spring *e* and spring *f* of the printed material are:

$$\varepsilon_{pe}(t) = \dot{\varepsilon}_T\gamma_{fe}(\frac{\gamma_0}{1-\gamma_0}\tau_{fr}(e^{-\frac{t}{\tau_{fr}}} - 1) - t) \tag{8}$$

$$\varepsilon_{pf}(t) = -\dot{\varepsilon}_T(\tau_{fr}(e^{-\frac{t}{\tau_{fr}}} - 1) + t) \tag{9}$$

Afterward, when temperature is lower than the glass transition temperature, the material behaves as elastic material which is governed as linear elastic Hooke's equation. The strains during this process are given as:

$$\varepsilon_{pe}(t) = -\alpha_p\dot{T}t\frac{1-\kappa}{1+\gamma_{ge}}, \quad \varepsilon_{pf}(t) = 0 \tag{10}$$

where $\gamma_{ge} = E_{ge}h_p / (E_s h_s)$ and E_{ge} is the elastic modulus of the spring *e* below T_g. Thus, the strain for the printed materials during the whole of cooling process is described by the sum of strain in equations (8)-(10).

As for the third process, after the material is cooled down to a room temperature, removing the printed material from the supporting platform leads to the recovery of elastic deformation (Fig. S1(c)), that is, the strain kept in the spring *e* will recover completely while the strain in the spring *f* will be confined since the relax time at the elastic state is long enough. Namely, a residual strain with the value of ε_r retains in the spring *f*:

$$\varepsilon_r = \varepsilon_{pf}(t_1) + \varepsilon_0 = -\alpha_p \dot{T}(1-\kappa)\gamma_0(\tau_{fr}(e^{-\frac{t_1}{\tau_{fr}}} - 1) + t_1) + \varepsilon_0 \qquad (11)$$

Therefore, the 3D-printed material can be considered to be a self-equilibrium state after being removed from the platform, where the spring *f* provides the recovery force while the dashpot provides the resistant force.

We consider the forth process that the printed sample is reheated again. When the printed PLA strip is heated with a heating rate $\dot{T}_+$, it will expand with thermal strain $\alpha_p \dot{T}_+ t$ at beginning and then behave as a viscoelastic material once its temperature exceeds T_g. At that state, since the spring *e* has been recovered, the model can be simplified to be the Voigt model, which consists of the spring *f* and the dashpot. Thus, the strain at temperature over T_g can be given as

$$\varepsilon_p^+(t) = \alpha_p \dot{T}_+ t - \varepsilon_r(1 - e^{-\frac{t}{\tau_f}}) \qquad (12)$$

Theoretical model for pattern transformation of printed lattice material

According to equation (12), a printed ring will contract when it is reheated initially over T_g. The contraction of rings in the lattice can result in tension forces at the connected points between adjacent rings. However, the boundaries of the whole lattice structure are unconstrained. As a result, the assumed tension forces need to be released to zero quickly by structural motion or deformation. At the beginning of heating process, the polymer has a long relaxation time so that only spring *e* in our model can be stretched under the tension forces. The rings have large stiffness (modulus of spring *e*) to resist the deformation at that state, the circular rings therefore move toward the center of the structure until the tension forces vanish. The whole structure behaves as contraction without pattern transformation of circular microstructure. Later, polymer is gradually softened and moduli of spring *e* and *f* in the model drop dramatically. As the reduction of stiffness of rings makes the rings easy to deform, the assumed tension force can trigger the deformation of the circular rings. Afterward, viscosity of the material related to the dashpot is more significant than elasticity, which makes the deformed shape fixed instead of elastic recovery, and the novel patterns are finally formed. For the sake of simplicity, a model of the curved beam is adapted to describe the deformation process, where an assumed concentrated tension force *F* is applied on the connected point of rings, while the other end is fixed due to the symmetry of the structure, as shown in Fig. S2. The deflection of the ring can be obtained according to Ref. 2:

$$\omega(\theta)=\frac{FR^3}{2D}f(\varphi,\theta) \tag{13}$$

where $f(\theta,\varphi)=(\varphi-\theta)\cos\theta-\cos\varphi\sin(\varphi-\theta)$ is shape function, θ is polar coordinate of the circular ring in the lattice structure and φ describes the lattice structure of the material, that is, $\varphi = \pi/6$ and $\pi/4$ corresponds to hexagonal and square lattice structure, respectively; R is radius of the ring; D is bending stiffness of the ring. As the deflection at the connected point is equal to $\varepsilon_p^+(t)R$, that is, $\omega(0)=FR^3f(\varphi,0)/2D=\varepsilon_p^+(t)R$, we obtain:

$$\omega(\theta)=\varepsilon_p^+(t)R\frac{f(\varphi,\theta)}{f(\varphi,0)} \tag{14}$$

and the deformed shape of the curved ring can be described as:

$$\omega(\theta)=R\left\{1+\varepsilon_p^+(t)\left[1-\frac{f(\varphi,\theta)}{f(\varphi,0)}\right]\right\} \tag{15}$$

Finally, the transformed shape of the printed lattice with time can be described approximately by equation (15) and is consistent with our experimental results.

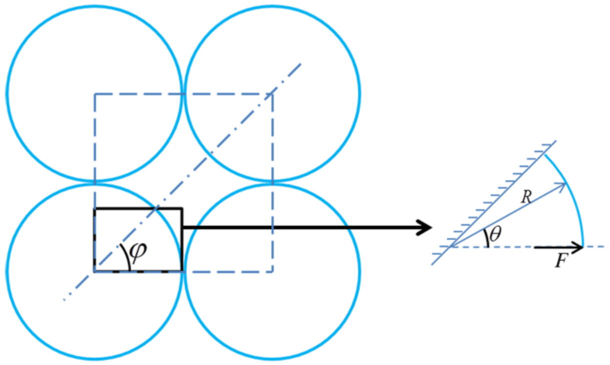

Figure S2 Schematic of lattice material fabricated by 3D printing technique.

1. Lin, J. R. & Chen, L. W. The mechanical-viscoelastic model and WLF relationship in shape memorized linear ether-type polyurethanes. *J. Polym. Res.* **6**, 35-40 (1999).
2. Timoshenko, S. & Gere, J. M. *Mechanics of Materials*, Van Nostrand Reinhold Co., New York, America (1973).
